# Establishment of reference values for selected haematological parameters in young adult Nigerians

**DOI:** 10.1371/journal.pone.0213925

**Published:** 2019-04-02

**Authors:** Ojor Ayemoba, Nurudeen Hussain, Tahir Umar, Anthony Ajemba-Life, Terfa Kene, Uchechukwu Edom, Ikechukwu Ogueri, Goodluck Nwagbara, Inalegwu Ochai, Chikwado Eneja

**Affiliations:** 1 Ministry of Defence Health Implementation Programme, Abuja, Nigeria; 2 Ave Health Sense Ltd, Area II, Abuja, Nigeria; University of Witwatersrand/NHLS, SOUTH AFRICA

## Abstract

**Background:**

Haematological reference values are necessary for accurate diagnosis of diseases, clinical decision-making, treatment monitoring and clinical research. Due to unavailability of pan-Nigerian reference values, local results interpretation is based on the use of Western values.

**Objective:**

This study aims to establish national reference values for some haematological parameters in apparently healthy young adult Nigerians.

**Methods:**

Seven Thousand, Seven Hundred and Ninety-Seven (7,797) volunteers aged 18 to 26 years participated in the study. Participants’ interviewer-administered questionnaires and blood samples were collected, analyzed and those with confounding factors (HIV infection, Hepatitis B sero-positivity, Malaria and Pregnancy) were excluded from statistical analysis. The 95-percentile reference range was determined for each haematological parameter using SPSS Version 16^®^. The result obtained was reviewed with reference to already established reference values in two West African and Western countries.

**Results:**

Blood specimens from 6153 (78.9%) participants [comprising 5915 (96.1%) males and 238 (3.9%) females] were analyzed after exclusion of 1,644 (21.1%) participants with confounding variables. Reference ranges among males and females varied and these were consistent with findings from two other West African countries. The median platelets count in this study was 218 x 10^9^/l while commonly used Western value is 280 x 10^9^/l. Other haematological parameters in the study were similar to Western values.

**Conclusion:**

The findings of this study will improve clinical and research decision-making. Considering that this study is limited to young adult participants, there is a need to conduct future pan-Nigerian studies that will include all age groups.

## Introduction

Laboratory reference values are necessary for clinical decision making in the hospital, identification of pathological changes, accurate interpretation of laboratory results and utilization for clinical research such as vaccine or other trials. However, these reference values differ based on many socio-demographic characteristics. Several factors such as age, gender, dietary patterns, ethnic differences and altitude affect the reference ranges for different groups [1]. Pre-analytical variables, laboratory processes and quality control in the laboratory may not change or influence reference ranges but could falsely alter individual test results [2]. Recent pan-Nigerian immuno-haematological and biochemical reference values are hardly available. Reference values validated in adult populations elsewhere could be misleading and may impact negatively on clinical management of patients. Studies from several African countries have shown variation in hematological parameters when compared with documented reference ranges validated in Western populations[3–7]. Similarly, within Nigeria, differences in hematological reference values have also been observed due to differences in the study population size, study area, and methodology [8–10].

Some studies reported reference values for all age groups without disaggregating by sex [2], while others disaggregated by sex but combined all age groups [8,9]. These present a limitation in application of the reference ranges in everyday clinical decision making. Similarly, hospital-based studies and those of specific target populations such as blood donors do not represent the general population [10–13]. Furthermore, most of the studies were restricted to some geographical sub-populations in Nigeria [8–11]. As such, Nigeria lacks recent reference values that are truly representative of its general healthy population. Thus, most haematological reference values used in Nigeria are derived from Western populations [10–12]. Establishment of reference values in Nigeria is a major challenge because of the huge size of the country with the attendant costs of large population studies, so several efforts are limited to small regional populations. Considering that this study involves all the states of the Federation, the findings will reflect a national spread for the age group 18–26 years. Therefore, this study seeks to determine the haematological reference values among young Nigerian adults applying for military service.

## Subjects and methods

### Study population

This cross-sectional study was carried out between March 2014 and October 2017 in the laboratories of 12 military hospitals spread across the 6 geo-political zones of Nigeria. The study population included seven thousand, seven hundred and ninety-seven (7,797) apparently healthy young Nigerian adults, aged 18–26 years, wishing to be enlisted for military service. Interviewer administered questionnaires were used on consenting participants to determine their socio-demographic characteristics such as age, gender, marital status, state of origin, level of education and employment status. This was followed by collection of blood samples for full blood count (FBC), Hepatitis B surface antigen (HBsAg), HIV-1 and 2, malaria and pregnancy testing. Rapid diagnostic tests were used to detect serological markers of infection or pregnancy. Assays were performed for the following haematological parameters: Red Blood Cell Count (RBCC), Haemoglobin Concentration (Hb), Packed Cell Volume (PCV), Mean Cell Volume (MCV), Mean Cell Haemoglobin (MCH), Mean Cell Haemoglobin Concentration (MCHC), Platelets Count (Plt), Total and Differential White Blood Cell Counts (WBCC).

### Specimen collection and rapid testing for biomarkers

About 4.5 mls of whole blood was obtained from each participant using ethylene-diamine-tetra-acetic acid (EDTA) vacutainer collection tubes (Becton Dickinson^®^, NJ, USA). Urine samples were collected for urinalysis using universal containers. All samples were analysed and processed within 3 hours of collection. Haematological analysis was performed on whole blood after thorough mixing using a blood mixer. Thereafter, plasma samples were extracted by centrifugation for serological testing. Rapid testing for HIV-1 & 2 were performed using the Nigerian National HIV serial testing algorithm (Determine^®^, Unigold^®^ and Stat-Pak^®^); HBsAg detection and pregnancy status using LabACON^®^ kits (Citus Diagnostic Inc^®^, British Columbia, Canada) and malaria infection using SD BIOLINE^®^ Rapid Diagnostic Test kit (Standard Diagnostics Inc^®^, Korea). Combi-9^®^ urinalysis rapid test kit (Machery-Nagel GmbH & Co-KG^®^, Duren, Germany) was used to detect haematuria. All assays were performed in accordance with product manufacturers’ guidelines.

### Haematological analysis and quality control

Haematological analysis (FBC) was carried out using Sysmex KX-21-N haematology auto-analyzer^®^ (Sysmex Corporation Inc^®^, Kobe, Japan). Due to the limited capability of Sysmex KX-21-N haematology auto-analyzer^®^, which is a 3-part differential platform, differentiation into Eosinophils, Basophils and Monocytes could not be achieved. The combined Eosinophil, Basophil and Monocyte counts were therefore reported as mixed cell count.

Laboratorians who have consistently passed Competency Testing for at least 3 years prior to study commencement were drawn from the internationally accredited Defence Reference Laboratory, Abuja, to carry out specimen analysis in the field. Routine Quality Control (QC) procedures such as equipment/assay validation and use of commercially prepared controls were strictly adhered to. Data confidentiality and quality were ensured through specimen de-identification/results aggregation and double entry by 2 independent Data Entry Clerks.

### Statistical analysis

Data sets were entered on a designed Excel template and exported to SPSS^®^ for analysis. Results of participants with the presence of antibodies against HIV and HBsAg in plasma were excluded from data analysis. Those who also showed presence of Plasmodium species or haematuria were equally excluded from the analysis. Results of female participants were excluded if they were found to be pregnancy test positive.

Being a descriptive study, measures of central tendency (median) and corresponding reference ranges were calculated for each haematological parameter. The 95^th^ percentile reference range was determined using 2.5 and 97.5 percentiles. Studies of ‘reference values’ in adult populations were searched in PubMed using relevant key words to review the similarities or otherwise of their published estimates with the results of this study.

### Ethical considerations

Ethical approval was obtained from the Ministry of Defence Health Research Ethics Committee (MODHREC). Written informed consent was obtained from each volunteer, followed by HIV pre- and post-test counseling. Participants who tested positive to bio-markers of infection were referred to the nearest care and treatment centre. Personal identifiers were eliminated through the use of unique Study Identification Numbers (SIN).

## Results

A total of 7,797 apparently healthy young people from age 18 to 26 years in all the states of Nigeria participated in the study. Of the number, 1,644 (21.1%) participants tested positive for HIV, HBsAg, Malaria parasite or pregnancy and were excluded from data analysis while 6,153 (78.9%) eligible participants [comprising 5915 (96.1%) males and 238 (3.9%) females] had their questionnaire responses and laboratory results statistically analyzed. The auto-analyzer could not provide the results for all parameters in all the specimens (N) leading to missing values for some parameters.

The results were segregated by gender for each parameter although no comparison was made and therefore no statistical test of significance was required. The sample size, median, 95 percentile reference values and gender-differentiated results are shown in Table 1. The findings in this study and Western values obtained from Dacie and Lewis Practical Haematology [14] are shown in Table 2, while Table 3 shows findings from this study and two neighboring West African countries [Ghana and Togo] [15,16].Median platelets count observed in this study (218 x 10^9^/l) was seen to markedly vary from Western Reference values (280 x 10^9^/l) (Table 2). Similarly, MCV was 84fl, 87fl and 85fl in this study and two other West African studies respectively [15, 16], while Western value was 92fl [14] (Tables 2 and 3). For the rest of the parameters, values from index study, neighboring Western African countries and Western values appeared similar.

**Table 1:** Statistical analysis of selected haematological reference parameters to establish normal reference values for adult nigerians between the ages of 16 and 26 years segregated by gender.

Test Parameters	Number of Participants tested (N)	Median	Reference Value (95% percentile)
**Red Blood Cell Count (x10**^**12**^**/l)** Male Female	5915 (96.1) 238 (3.9)	5.5 5.5	4.4–7.0 4.4–6.8
**Haemoglobin Concentration (g/l)** Male Female	5915 (96.1) 237 (3.9)	148 146	120–172 117–170
**Packed Cell Volume or Haematocrit (l/l)** Male Female	5915 (96.1) 237 (3.9)	0.46 0.46	0.37–0.53 0.38–0.54
**Mean Cell Volume (fl)** Male Female	5915 (96.1) 237 (3.9)	84.0 84.1	70.0–96.0 69.2–95.0
**Mean Cell Haemoglobin (pg)** Male Female	5915 (96.1) 237 (3.9)	27.0 27.0	21.0–31.5 21.0–31.2
**Mean Cell Hb Concentration (g/l)** Male Female	5915 (96.1) 237 (3.9)	320 320	290–350 280–348
**Platelet Count (x10**^**9**^**/l)** Male Female	5883 (95.6) 237 (4.4)	218.0 217.0	89.0–374.0 101.0–425
**Total White Blood Cell Count (x10**^**9**^**/l)** Male Female	5915 (96.1) 238 (3.9)	5.9 5.9	3.4–9.6 3.6–10.3
**Differential White Cell Count (x10**^**9**^**/l)** Neutrophils (Male) Neutrophils (Female) Lymphocytes (Male) Lymphocytes (Female) Mixed Cells (Male) Mixed Cells (Female)			
	5711	2.6	1.2–5.6
	230	2.7	1.3–6.0
	5915	2.4	1.1–4.3
	238	2.3	1.2–4.3
	5705	0.7	0.2–2.2
	230	0.7	0.2–2.3
**Differential White Cell Count (%)** Neutrophils (Male) Neutrophils (Female) Lymphocytes (Male) Lymphocytes (Female) Mixed Cells (Male) Mixed Cells (Female)			
	5711	45.5	26.0–69.0
	230	47.8	26.0–70
	5915	41.0	21.0–59
	238	40.5	22.0–60
	5705	12.0	3.0–29.0
	230	11.1	3.0–28

**Table 2:** Median haematological reference values from this study and western haematological reference values.

Test Parameters	This Study (Median)	Western Values*
**Red Blood Cell Count (x10**^**12**^**/l)** Male Female	5.5 5.5	5.0 4.3
**Haemoglobin (Hb) Concentration (g/l)** Male Female	148 146	150 135
**Packed Cell Volume (PCV) or Haematocrit (l/l)** Male Female	0.46 0.46	0.45 0.41
**Mean Cell Volume (fl)** Male and Female	84.0	92.0
**Mean Cell Haemoglobin (pg)** Male and Female	27.0	29.5
**Mean Cell Haemoglobin Concentration (g/l)** Male and Female	320	330
**Platelet Count (x10**^**9**^**/l)** Male and Female	218	280
**Total White Blood Cell Count (x10**^**9**^**/l)** Male and Female	5.9	N/A**

* Western Haematological values from Bain BJ *et al*[14]

** Median value not available, however, the reported reference range is 4.0–10.0 x 10^9^/l [14]

**Table 3:** Median haematological reference values from this study and two west African Countries.

Test Parameters	This Study	Ghanaian Values*	Togolese Values*
**Red Blood Cell Count (x10**^**12**^**/l)** Male Female	5.5 5.5	4.8 4.3	5.0 4.5
**Haemoglobin (Hb) Concentration (g/l)** Male Female	148 146	139 123	151 130
**PCV or Haematocrit (l/l)** Male Female	0.46 0.46	0.42 0.37	0.43 0.38
**Mean Cell Volume (fl)** Male and Female	84.0	87	85
**Mean Cell Haemoglobin (pg)** Male and Female	27.0	28.6	29.5
**Mean Cell Hb Concentration (g/l)** Male and Female	320	331	351
**Platelet Count (x10**^**9**^**/l)** Male and Female	218	216	239
**Total White Blood Cell Count (x10**^**9**^**/l)** Male and Female	5.9	5.4	4.1

*Ghanaian and Togolese values obtained from Dosoo*et al* [15] and Kuevakoe*et al* [16] respectively

## Discussion

Like many other African countries, most Nigerian health facilities often rely on laboratory reference values derived from Western populations for results interpretation [17, 18]. Inter-regional, inter-country and inter-racial differences in haematological reference values are well documented [3–7, 9, 18–20]. These studies show marked variation between African and Western values with African values being generally lower than Western values. Our Pan-Nigerian study findings are in agreement with these observations, except for RBCC, Hb concentration and PCV values, which are similar to Western values.

Many studies have shown that values for red cell parameters such as RBCC, Haematocrit, Hb and red cell indices like MCV, MCH and MCHC are higher in males than females [6, 9, 21, 22]. Such gender differences have been attributed to menstrual blood loss in females and androgenic hormonal influences in males among others, [6, 20, 22, 23, 24] with Hb levels being lowered in females through haemodilution while testosterone increases RBCC levels in males. The findings of this study (Table 1) did not show such gender disparity. This may be attributed to the fact that females applying for military service are likely to be athletic and less overweight than other women in the general population. Since low body fat is associated with low levels of estrogen [25, 26], this group of females may suffer less from the marrow suppressive effects of estrogen.

The median values for MCV, MCH, MCHC and Platelets count are lower than those of Western populations (Table 2) which is consistent with the findings of others [6, 9, 21, 22]. The lower haematological reference values among Africans when compared with Western values have been attributed to factors such as low dietary iron intake, higher prevalence of genetic polymorphisms such as sickle cell disorder, and endemic parasitic infections like malaria, hookworm, schistosomiasis, and other chronic infections [18]. These confounding factors were largely excluded from this study population, thus the similarity of our RBCC, Hb and PCV values to Western values may be a result of the minimization of such confounders.

This pattern is also reflected in the comparison of our findings with the West African countries of Ghana and Togo (Table 3). Slight intra-regional differences were observed. The RBCC, Hb, and PCV values of this study appeared to be higher while MCV, MCH, MCHC, Platelets count and total WBCC values were similar to those of the 2 West African countries. Diet, environmental and genetic factors have been cited as possible reasons for the observed differences but the true causes of intra-regional variations are largely unknown [27]. The observed lower reference values for Platelets count among Africans should always be considered in clinical interpretation and decision-making especially when conducting clinical trials, bone marrow or other organ transplantation and during administration of chemotherapy or radiation therapy, because of the marrow suppressive effects of some of these procedures with the possibility of thrombocytopenia-induced bleeding.

There were no remarkable differences between Nigerian WBC counts and Western or other compared values despite the well acknowledged high prevalence of chronic bacterial, viral and parasitic infections in Africa [6,15]. Eosinophil, Basophil and Monocyte counts were merged as ‘mixed cells’ because the Sysmex auto-analyzer^®^ used for the study was a 3-part differential model that could not segregate all the white cell types. A manual differential white cell counting method was not utilized in order to avoid inter-mingling of automated analyzer results with manually generated ones, as this may have quality assurance implications.

### Study limitations

Body Mass Index (BMI) and assessment for common parasitic infections like hookworm and schistosomiasis was not done even though both are known to influence normal values among endemic populations [6,15]. Rapid testing for hematuria was however performed to minimize the number of participants with possible schistosomiasis. Information about smoking, alcohol consumption and dietary pattern was not obtained even though efforts were made to take samples in the morning and before any physical activity. The findings in this study are applicable to young adults and do not cover all age groups.

## Conclusion

This pan-Nigerian study was able to establish haematological reference values in young healthy adult Nigerians. The current study has shown that reduction of confounding factors such as chronic infections, results in local African haematological reference ranges that are close to those of Western values. These findings are unique, and are relevant for clinical and research decision making in young Nigerian adults. They will also be useful as reference material and basis for the need to conduct similar pan-Nigerian studies that will include all age-group strata in future.
